# Universality in boundary domain growth by sudden bridging

**DOI:** 10.1038/srep21110

**Published:** 2016-02-22

**Authors:** A. A. Saberi, S. H. Ebrahimnazhad Rahbari, H. Dashti-Naserabadi, A. Abbasi, Y. S. Cho, J. Nagler

**Affiliations:** 1 Department of Physics, University of Tehran, P.O. Box 14395-547,Tehran, Iran; 2 School of Physics and Accelerators, Institute for research in Fundamental Science (IPM) P.O. 19395-5531, Tehran, Iran; 3 School of Physics, Korea Institute for Advanced Study, Seoul 130-722, Korea; 4 Department of Physics, Shahid Beheshti University, Evin, Tehran 19839, Iran; 5 Department of Physics, Plasma and Condensed Matter Computational Laboratory, Azarbaijan Shahid Madani University, Tabriz 53714-161, Iran; 6 Physics and Accelerators Research School, NSRTI 11365-3486, Tehran, Iran; 7 Department of Physics and Astronomy, Seoul National University, Seoul 151-747, Korea; 8 Computational Physics, IfB, ETH Zurich, Wolfgang-Pauli-Strasse 27, 8093 Zurich, Switzerland

## Abstract

We report on universality in boundary domain growth in cluster aggregation in the limit of maximum concentration. Maximal concentration means that the diffusivity of the clusters is effectively zero and, instead, clusters merge successively in a percolation process, which leads to a sudden growth of the boundary domains. For two-dimensional square lattices of linear dimension *L*, independent of the models studied here, we find that the maximum of the boundary interface width, the susceptibility *χ*, exhibits the scaling *χ* ~ *L*^*γ*^ with the universal exponent *γ* = 1. The rapid growth of the boundary domain at the percolation threshold, which is guaranteed to occur for almost *any* cluster percolation process, underlies the the universal scaling of *χ*.

Universality is an important concept in statistical physics which implies that the critical exponents characterizing the critical transition do not depend on the microscopic details of the model^1^. Percolation on lattices describes the sudden emergence of a spanning cluster together with its fluctuations. In site percolation in euclidean lattices the order parameter, usually defined as the fraction of occupied sites in the spanning cluster, is studied as a function of the control parameter *p* (the fraction of occupied sites of the entire lattice). The percolation universality class is characterized by a given set of critical exponents that determine the scale invariant behavior immediately before, precisely at and just after the phase transition from microscopic to global connectedness^2^,^3^,^4^,^5^. However, the scaling and hyperscaling relations leave only two independent exponents, e.g., *β* and *v* characterizing the critical behavior of the order parameter and the correlation length around the critical threshold 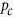, respectively, which fully determine the percolation universality class. The universality, on the other hand, can be encoded by the rich fractal structure of the percolation clusters at criticality. A fractal percolation cluster of fractal dimension 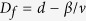, where *d* is the dimension of the system, is composed of several other fractal substructures including its perimeter (hull), external perimeter, backbone, and red sites (bonds), etc. For instance, it is shown^6^ that the fractal dimension 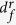 of the red bonds (a red bond is one that upon cutting leads to a splitting of the cluster) is given by 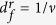 valid in all dimensions *d* below the critical dimension *d*_*c*_ at which the mean field exponents hold. Therefore, the universality can alternatively be given by the fractal geometry of the model in terms of 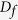 and 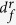.

In contrast to cluster aggregation processes at low cluster concentration, boundary domain growth in the limit of maximum concentration is poorly understood^7^,^8^.

Whereas at low concentration a cluster performs a random motion until it collides with another cluster or the boundary^9^,^10^,^11^,^12^,^13^, at high concentration the diffusivity is negligible and the process is well described by percolation. Here, we analyze a variety of percolation processes and ask how a given rule determines the growth of boundary domains.

The simplest of such processes is site percolation which can be considered a particular model for cluster-size dependent aggregation at maximal concentration. To demonstrate the universality of our framework we study a wide range of models and find that *all* models exhibit the same scaling of the susceptibility


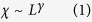


with the exponent, 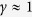. This universality is remarkable because other observables such as the fractal cluster dimension and the fractal surface dimension at the percolation threshold remain model specific.

## Results

We perform extensive Monte-Carlo simulations of cluster percolation on the square lattice with linear dimension *L*. Initially all lattice sites represent single clusters of unit size. Only neighboring clusters can merge each time step according to a given rule. Specifically, choose at each time step a cluster and merge the cluster according to a given rule with one of its neighboring clusters, accessible in its von Neumann neighborhood. Repeat this over and over again until a single cluster of size *N* spans the entire lattice.

**Figure 1:**
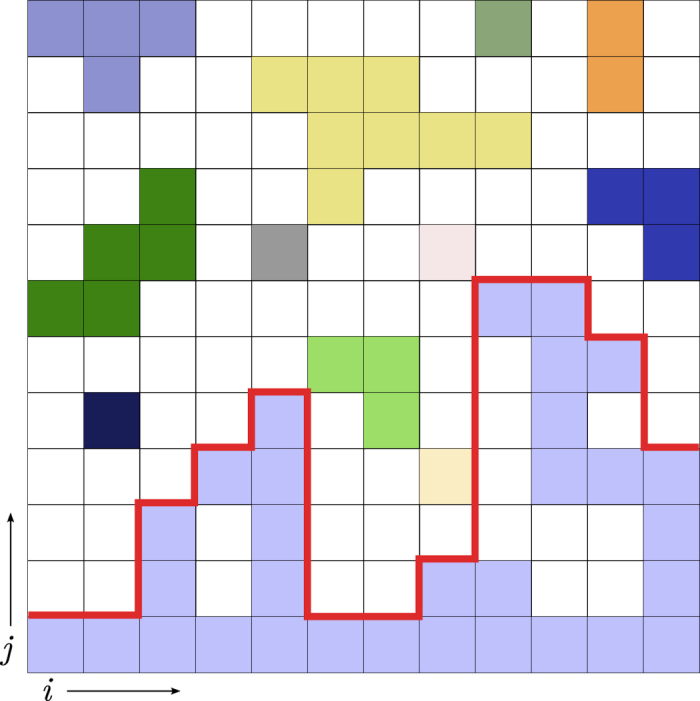
Schematic of the boundary domain. The (bottom) boundary domain consists of a single cluster (light blue) that evolves by merging with other neighboring clusters from the initial set of the *L* bottom sites (*i* = 0 to 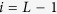; *j* = 0). The red line shows the height profile of the bottom boundary. Other clusters are color coded. White cells are isolated single-site clusters (equivalent to unoccupied sites in site percolation).

During the aggregation process, the system undergoes a phase transition from a subcritical phase of microscopic 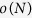-size components to a supercritical phase with (at least) a macroscopic component of size 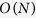. Here we analyze the growth of the lower boundary domain. In the beginning, the domain is a single cluster of size *L* which merges during the percolation process with other clusters at its interface, as sketched in Fig. 1.

We study models of different universality classes: (i) standard continuous site percolation, and models of discontinuous cluster percolation (ii–v). (Dis)continuity refers to the behavior of the order parameter at the critical percolation threshold. Specifically, for models (ii–v), at each step a cluster is selected uniformly at random, independent of its size, and (ii) merged with its smallest neighbor cluster, referred to as min-rule, (iii) merged with its largest neighbor cluster (max-rule), or (iv) merged with a randomly selected neighbor cluster (rnd-rule). To further demonstrate the universality of our findings, we also study the recently introduced (v) “fractional percolation” rules where the merging of clusters with substantially different sizes is systematically suppressed and components are preferentially merged whose size ratio is close to a fixed target ratio, *f*. As a result, the order parameter displays discontinuous jumps reminiscent of the crackling noise^14^. Note that all these models cover very different aggregation processes in the limit of maximal density. Other ‘explosive’ percolation models, which were proven to be continuous, though exhibiting a substantial gap in the order parameter for large finite systems, do not show universality, meaning each microscopic connection rule defines its own universality class. Rules (ii–v) are truly discontinuous percolation models, and thus cannot be related (such as via a set of critical exponents) to standard universality classes of (continuous) percolation. The main reason why we choose those ‘exotic’ models is to have a broad spectrum of very different percolation processes.

In the models a neighboring cluster refers to von-Neumann neighborhood of boundary sites, and cylindrical (half periodic boundary) conditions are applied. In order to account for size-dependent delay for processes (ii–v), after each merger, time is advanced by 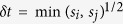 where 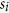 and 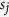 are the respective relative sizes of the merging clusters (other choices do not affect any of the conclusions)^14^.

**Figure 2:**
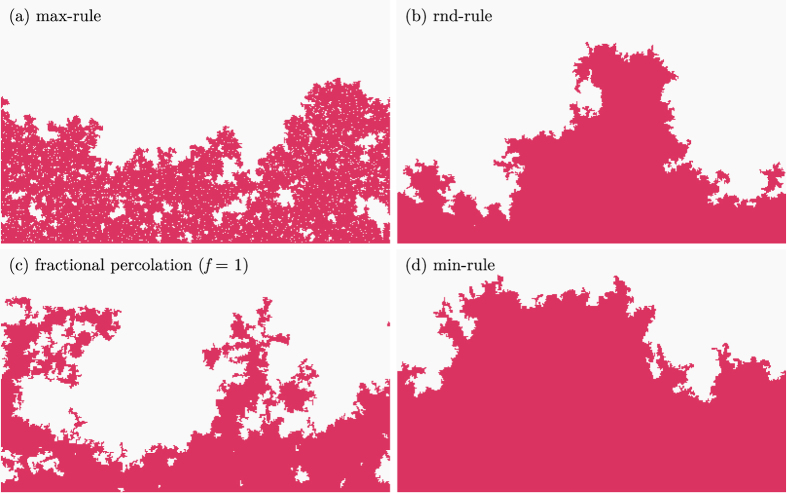
Subcritical boundary domains. Snapshots of the growing boundary domain for different models exactly one step *δt* before percolation. (**a**) max-rule produces a very porous and loose boundary domain. (**b**) rnd-rule generates a dense and space-filling cluster (fractal dimension 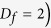. (**c**) fractional percolation 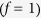 exhibits an almost compact boundary domain. (**d**) min-rule shows a compact boundary domain without voids inside its bulk. All models on square lattice of size 400 × 400; shown is the lower domain of size 400 × 250.

Typical snapshots of the growing boundary domain for the (a) max-rule, (b) rnd-rule, (c) fractional, and (d) min-rule demonstrate that the roughness and the porosity of the boundary are strongly dependent on the growth mechanism (Fig. 2).

For min-rule (ii) and rnd-rule (iv) the critical boundary domain cluster is a compact surface fractal, meaning the fractal dimension of the boundary cluster, 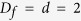 (in the thermodynamic limit), where *d* is the lattice dimension. This implies a genuine discontinuity of the percolation phase transition^15^,^16^. For other models, the fractal dimension of the boundary domain is close to 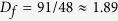 characteristic of the standard percolation universality class in two dimensions (see Supplementary Information). The universality of Eq. (1) holds regardless whether or not the critical percolation cluster is fractal or compact (or whether or not the cluster surface is smooth or fractal). To demonstrate this, we study the roughness of the surface of the growing boundary characterized by the rms (root mean square) fluctuation of heights, *w*,


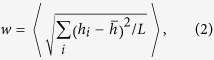


where 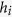 are the height of the boundary domain at boundary positions 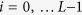, and mean 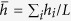, see Fig. 1.

**Figure 3:**
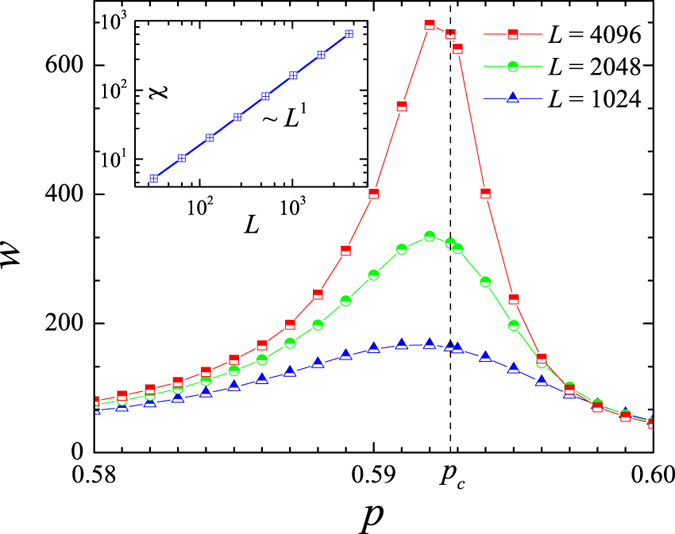
Roughness of the boundary domain in site percolation. The rms fluctuations of height, *w*, as a function of occupation probability *p* (equivalent to time using kinetic formulation, i.e. *t* = *p* for ordinary percolation). Inset: Scaling of the susceptibility with size. Square lattices of size *L*, 10^5^ realizations. Error bars are smaller than symbol size.

**Figure 4:**
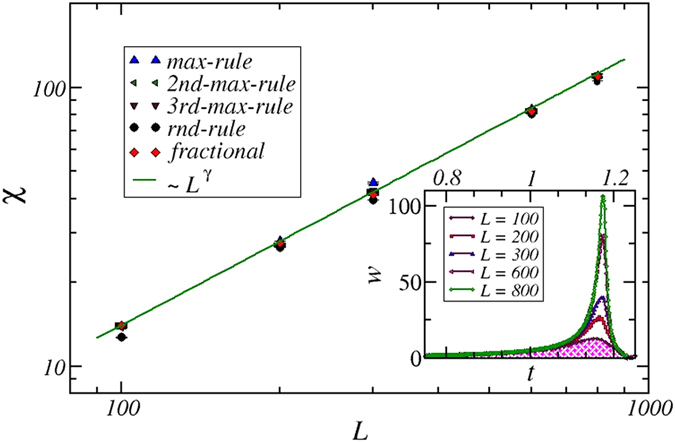
Universality of the susceptibility scaling. Inset: interface width, *w* (Eq. (2)), for rnd-rule as a function of time for different lattice size *L*. Main panel: Maximum of the interface width, the susceptibility, Eq. (1), at the percolation point, as a function the lattice size *L* for max-rule (Δ), 2nd-max-rule (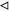), 3rd-max-rule (∇), rnd-rule (○), and fractional 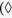, *f* = 1.0). The solid line shows the best fit, 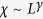, where 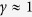 (max-rule: 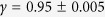, 2nd-max-rule: 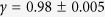, 3rd-max-rule: 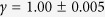, rnd-rule: 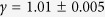, fractional: 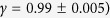. 800 realizations for each data point.

The rms fluctuation of heights, *w*, exhibits a peak at the percolation point, 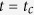 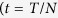 is the scaled time, *T* denoting the MC steps), as shown for model (i) in Fig. 3. Most remarkably, the susceptibility, *χ*, defined as the maximum of *w*,


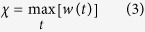


increases with lattice side length, 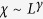, independent of the models used here, with the exponent, 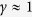 (Fig. 4).

In diffusion limited aggregation, for example, microscopic particles diffuse until they touch other particles or the boundary. Such processes are thus characterized by a continuous growth of the boundary domain, where usually 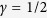. In contrast, through rules (i–v) a successive aggregation of clusters that cannot move and are initially nearest neighbors is studied. In case (i) this aggregation is known as ordinary site percolation. Since ordinary site percolation exhibits a rapid but continuous emergence of a unique giant cluster exactly at *p*_*c*_, and all other clusters are of size 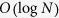 before and after 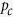, the naive expectation would be that boundary growth may also be continuous (thus characterizing by some 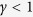, if not 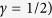. In addition, the fractal geometry of the giant percolation cluster and its boundary do depend on the model^3^,^8^,^16^ (see Supplementary Information). So, a universal 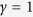 is a rather surprising finding. In the following, we explain the universality by the necessary occurrence of a sudden bridging.

**Figure 5:**
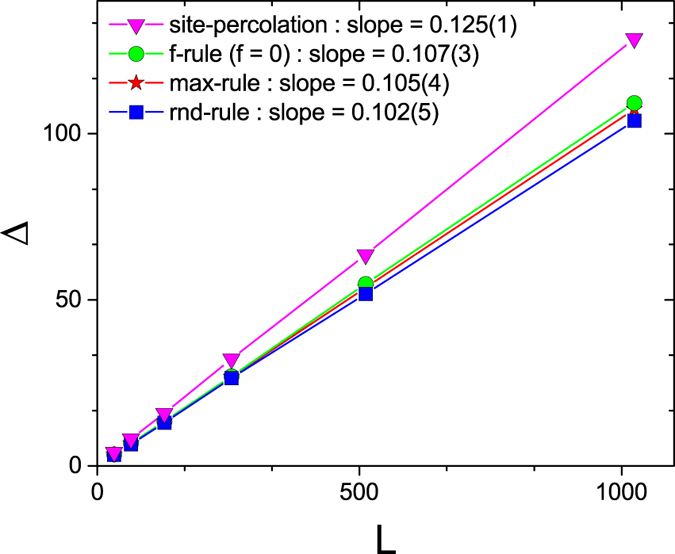
The maximal gap in *w*. Size Δ of the largest gap in *w* for a collection of continuous and discontinuous cluster percolation models. Specifically, for rnd-rule 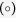, 2nd-max-rule (W), 3rd-max-rule 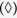, fractional (Δ, 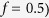, all yielding discontinuous percolation, and max-max-rule (select at random a cluster and merge the two largest clusters that are neighbors of each other among the selected cluster and all its neighbors), yielding continuous percolation, Δ as a function of lattice size *L* is shown. 800 realizations for each data point. Error bars are smaller than symbol size.

Consider the largest single step jump in *w*,


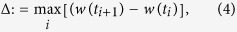


which occurs at the percolation point, *t*_*c*_, as shown in Fig. 5.

Because the spanning cluster is macroscopic, *i.e.*, of size 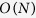, the linear dimension of the percolation cluster is of size 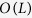, in *any* linear dimension. Thus, at percolation an 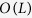 number of boundary sites 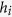 jump from 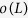 to 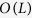.

**Case 1:** *αL* number of sites 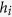 increase to 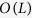, and 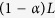 sites stay of size 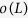, with some 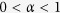. Then the mean difference 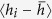 is of size 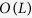, resulting from the 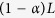 fraction of sites that have an 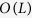-sized difference to 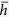. Thus 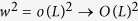.

**Case 2:** Assume 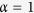, meaning all sites jump from 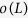 to 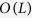. Unless the spanning cluster exhibits only 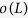 fluctuations parallel to the boundary domain, the mean difference 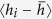 is of size 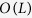. Recall that the linear dimension of the percolation cluster is of size 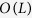 in *any* direction, in particular parallel to the height profile 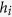. Thus 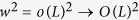.

**Case 3:** All sites jump from 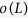 to 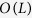 *and* the height profile of the spanning cluster exhibits only 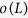 fluctuations parallel to the boundary domain. In this case, 
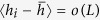
, with a possible 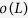 fraction of sites that show variations of size 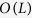. This determines the spanning cluster not only necessarily compact (characteristic of discontinuous percolation) but rectangular (possibly with “micro-cracks“). This very special case does not show a macroscopic jump in *w*.

We conclude that bridging implies 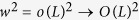 and thus 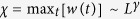 with 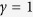, virtually independent of the model.

## Discussion

**Figure 6:**
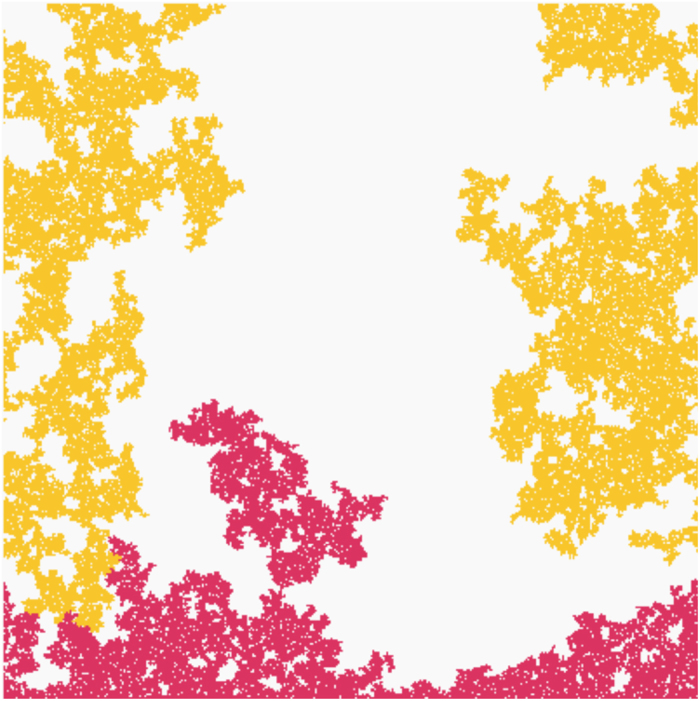
Sudden bridging. The fractal boundary domain (bottom, red) suddenly gets connected to the spanning cluster (yellow). This sudden event represents case (3) and induces a discontinuity in the domain growth leading to *γ* = 1.

In continuous percolation, the emergence of a unique macroscopic cluster necessarily coincides with the occurrence of spanning (when facing sides of the lattice get connected by a path of sites). At the percolation threshold the giant component is fractal and spanning. Discontinuous percolation, however, can show a much richer dynamics than case (3)^14^,^15^,^16^,^17^,^18^,^19^,^20^,^21^,^22^. In discontinuous percolation the emergence of a macroscopic cluster must not necessarily coincide with the emergence of a spanning cluster, nor must the giant component be unique at percolation. Instead, multiple giant (compact) components can emerge simultaneously^23^,^24^, which may merge in multiple discontinuous transitions. Spanning can occur much later than the first emergence of the macroscopic component. Nevertheless, as illustrated in Fig. 6, there necessarily occurs a single event where one of the 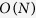-size components connects to the boundary domain, yielding an 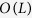-size jump in *w*. This predicts 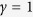 for both continuous and discontinuous processes, except for very particular processes.

We call those processes *needle growth processes*: With sufficient preference choose mergers such that the aspect ratio of the cluster that results from the merging is as large as possible. This rule (and other artificially constructed rules) would lead to spanning prior to the emergence of a macroscopic cluster. Thus the boundary domain would increase continuously in the thermodynamic limit.

Notably, processes where the emergence of a macroscopic cluster proceeds spanning are also possible: With sufficiently large preference grow the second largest cluster in the system such that its aspect ratio stays as close as possible to unity. This guarantees the simultaneous emergence of two macroscopic 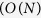-size) compact clusters reluctant to span the lattice at the percolation threshold (defined via the first emergence of a macroscopic cluster and not via spanning).

However, boundary domain growth for those processes would still exhibit an 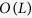-size jump in *w* because spanning is certain at times during the process.

Continuous domain growth is not only expected for *needle processes* but known for a broad class of physical relevant processes. Examples include, percolation or aggregation processes where boundary growth is the dominating process such as in invasion percolation or KPZ growth models^25^. More specifically, classification of the evolution of (1 + 1)-dimensional boundary domains in non-equilibrium growth processes has been very well established in the past^26^. One of the most important universality classes is given by the Kardar-Parisi-Zhang (KPZ)^25^ equation 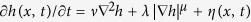 with 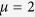, which also includes the Edwards-Wilkinson (EW) universality for 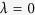. The boundary fluctuations reach a maximum *χ* in the stationary state which scales with the system size as 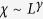. It is shown^27^ that in the presence of the additive noise *η*, the roughness exponent *γ* falls into the ordinary KPZ (EW) class with the exact value^25^ 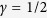 for all *μ* (*λ* = 0). However, for the deterministic case of *η* = 0 and for 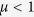, an instability occurs which leads to a fluctuating grooved interface. In this case, the roughness exponent is observed to coincide with our prediction 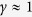
27.

To conclude, boundary domain growth at maximal concentration is discontinuous and characterized by a universal exponent with respect to the scaling of the maximum of the boundary interface width. The universality for boundary domain growth at maximal concentration in terms of the model-independent exponent 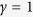 is explained by the necessary occurrence of sudden bridging, the connection of the boundary domain to the largest cluster in the system. Our study opens a new category of growing interfaces complementary to the well-established self-affine surfaces. Loosely speaking, in the non-isotropic *self-affine* growing interfaces, the exponent *γ*, which determines the universality class of the growth process, is model specific while the fractal properties of the boundary domain and its surface (if any) do not have any information about the universality class. In our case, the story is rather inverse: for an isotropic *self-similar* growing interface, the fractal structure is model specific characterizing the universality classes (if any), while the exponent 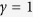 is super-universal for all models. In this picture, the exponent *γ* captures the underlying isotropic symmetry in the growth processes.

We found a universal scaling behavior of an important observable across a wide range of percolation models (i.e., for discontinuous and continuous percolation) that has not been reported as of yet: a universal scaling of the boundary domain growth induced by a phenomenon which we call sudden bridging. Previous aggregation models (i.e., diffusion limited aggregation) assume that microscopic particles diffuse until they collide with other particles (or the boundary), which usually leads to 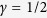 (and not 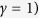. In a broader context as an empirical application of our finding, it is worth noticing that one of the crucial aims in surface growth science is to devise a dynamical growth model and mechanisms to understand the underlying physics behind the observed height profile in the lab using different tools, e.g., Atomic Force Microscopy (AFM). In AFM sample scans, the tip which moves along a 1d sample, only sees effective columnar valleys regardless of the inherent complex fractal structure of the grown surface a little deep inside. In this light, our study suggests that different percolation-based growth processes with different characteristic complex inherent structures can lead to the same statistics observed at the effective surface of the samples. To our knowledge, such correspondence has never been reported yet.

## Methods

We perform large scale Monte-Carlo simulations on a 2*D* square lattice of length *L*. Periodic boundary conditions are applied along the horizontal *x*-direction. We start with 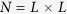 single clusters (meaning at 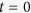 each site represents an individual cluster).

At each time step, we merge two neighboring clusters according to a fixed rule. We choose von Neumann neighborhood (i.e. given by either 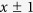, or 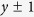; sites or clusters with a double displacement in *x* and in *y* direction are no neighbors).

At each MC step the number of clusters in the system decreases by 1 and eventually at the end of simulations one cluster emerges which then covers the entire lattice.

We use two different markers to identify bulk and domain clusters. At the beginning of the simulations, all the sites (and clusters) of the first row of the grid (at 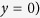 are marked black while the rest are white. Hence, the boundary domain at 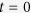 constitutes of *L* clusters at 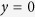 whereas the bulk constitutes 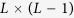 clusters in the domain 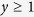. Whenever a bulk cluster (marked in white) is merged with a domain cluster (marked in black) it will join the domain. As time advances, the interface at the bottom will experience an upward directed but stochastic growth. The percolation time, 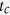, is defined through the MC step at which the boundary domain touches the ceiling of the system (at 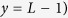, usually referred to as spanning.

Except for standard site percolation, we study two models types: (i) *Focal models*: For focal models we choose randomly a cluster *(focal cluster)* and merge it with one of its von Neumann neighboring *clusters*. Specifically, max-rule means choose at random a cluster and merge it with the largest neighboring cluster, min-rule means choose at random a cluster and merge it with the smallest neighboring cluster and for rnd-rule we choose at random a cluster and merge it with randomly chosen neighboring cluster. For the *fractional rule* choose at random a cluster and merge it with the von Neumann neighboring cluster (nn) that minimizes 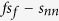 where 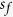 is the size of the focal cluster, 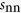 the size of the neighboring cluster and *f* a constant (a parameter of the model that controls to what extent clusters of a certain size ratio merge preferentially together^14^). Models based on focal kernels thus necessary involve the growth of the randomly chosen focal cluster.

In contrast, we study also (ii) *non-focal models* where the focal cluster does not necessarily aggregate with some other at a given MC step: choose at random a cluster, independently of its size (call this cluster the *focal cluster*). The focal cluster will be surrounded by other clusters (call these clusters *neighboring clusters*), which share at least a single von Neumann neighboring site (i.e. coordinate displacements 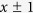, or 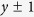 define the neighboring). Now consider the set 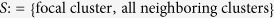. Merge two clusters of the set *S* that are neighboring, according to some given fixed rule (merging two clusters in *S* that are not neighbors is forbidden). Specifically, max-max rule: choose at random a cluster, call it focal cluster, find the largest cluster in the set *focal cluster plus all von Neumann neighbors* and merge it with its largest neighbor cluster. 2nd-max rule: choose at random a cluster, find the second largest cluster in the set *focal cluster plus all von Neumann neighbors* and merge this cluster with its largest neighbor. 3rd-max rule: choose at random a cluster, find the third largest cluster in the set *focal cluster plus all von Neumann neighbors* and merge this cluster with its largest neighbor.

## Additional Information

**How to cite this article**: Saberi, A. A. *et al.* Universality in boundary domain growth by sudden bridging. *Sci. Rep.* **6**, 21110; doi: 10.1038/srep21110 (2016).

## Supplementary Material

Supplementary Information
