# Corrigendum: Universality in boundary domain growth by sudden bridging

**DOI:** 10.1038/srep25560

**Published:** 2016-06-23

**Authors:** A. A. Saberi, S. H. Ebrahimnazhad Rahbari, H. Dashti-Naserabadi, A. Abbasi, Y. S. Cho, J. Nagler

Scientific Reports 6: Article number: 21110;10.1038/srep21110 published online 02222016; updated: 06232016

The original version of this Article contained errors.

H. Dashti-Naserabadi and A. Abbasi were incorrectly listed as being affiliated with ‘Department of Physics, Plasma and Condensed Matter Computational Laboratory, Azarbaijan Shahid Madani University, Tabriz 53714-161, Iran’ and ‘Physics and Accelerators Research School, NSRTI 11365-3486, Tehran, Iran’ respectively. The correct affiliations are listed below:

H. Dashti-Naserabadi

Physics and Accelerators Research School, NSRTI 11365-3486, Tehran, Iran

A. Abbasi

Department of Physics, Plasma and Condensed Matter Computational Laboratory, Azarbaijan Shahid Madani University, Tabriz 53714-161, Iran

In the Abstract,

“The rapid growth of the boundary domain at the percolation threshold, which is guaranteed to occur for almost *any* cluster percolation process, underlies the the universal scaling of *χ*.”

now reads:

“The rapid growth of the boundary domain at the percolation threshold, which is guaranteed to occur for almost *any* cluster percolation process, underlies the universal scaling of *χ*.”

In the legend of Figure 1,

“The (bottom) boundary domain consists of a single cluster (light blue) that evolves by merging with other neighboring clusters from the initial set of the *L* bottom sites (*i* =  0 to *i* =  *L* −  1; *j* =  0).”

now reads:

“The (bottom) boundary domain consists of a single cluster (light blue) that evolves by merging with other neighboring clusters from the initial set of the *L* bottom sites (*i* = 0 to *L* - 1; *j* = 0).”

In the legend of Figure 5,

“Size Δ of the largest gap in *w* for a collection of continuous and discontinuous cluster percolation models. Specifically, for rnd-rule (○ ), 2nd-max-rule (W), 3rd-max-rule (◇), fractional (Δ, *f* =  0.5), all yielding discontinuous percolation, and max-max-rule (select at random a cluster and merge the two largest clusters that are neighbors of each other among the selected cluster and all its neighbors), yielding continuous percolation, Δ as a function of lattice size *L* is shown. 800 realizations for each data point. Error bars are smaller than symbol size.”

now reads:

“**The maximal gap in *w***. Size Δ of the largest gap in *w* for a collection of continuous and discontinuous cluster percolation models. Specifically, for fractional *f*= 0 (∘), max-rule (☆), rnd-rule (□), all yielding discontinuous percolation, and site-percolation (▿), yielding continuous percolation, Δ as a function of lattice size *L* is shown. 10^5 realizations for each data point. Error bars are smaller than symbol size.”

These errors have now been corrected in the PDF and HTML versions of the Article.

