# Supplementary material for: Universality in boundary domain growth by sudden bridging
Source: Sci Rep. 2016 Feb 22;6:21110. doi: 10.1038/srep21110 (PMC4761969; doi:10.1038/srep21110)
Supplement: Supplementary Information [file srep21110-s1.pdf]

Universality in boundary domain growth by sudden bridging - Supplementary Information

A. A. Saberi[1,2], S. H. Ebrahimnazhad Rahbari[3,4], H. Dashti-Naserabadi[1], A. Abbasi[5], Y. S. Cho[6], and J. Nagler[7]
[1]*Department of Physics, University of Tehran, P.O. Box 14395-547,Tehran, Iran*
[2]*School of Physics and Accelerators, Institute for research in Fundamental Science (IPM) P.O. 19395-5531 , Tehran, Iran*
[3]*School of Physics, Korea Institute for Advanced Study, Seoul 130-012, Korea*
[4]*Department of Physics, Shahid Beheshti University, Evin, Tehran 19839, Iran*
[5]*Department of Physics, Plasma and Condensed Matter Computational Laboratory, Azarbaijan Shahid Madani University, Tabriz P.O. 53714-161,Iran*
[6]*Department of Physics and Astronomy, Seoul National University, Seoul 151-747, Korea and*
[7]*Computational Physics, IfB, ETH Zurich, Wolfgang-Pauli-Strasse 27, 8093 Zurich, Switzerland*

In this supplementary material, we will present some additional details of simulations and the results reported in the paper. It includes the study of the ordinary (site and bond) percolation problems together with the details of computations for the scaling properties of critical clusters.

I. STANDARD PERCOLATION MODELS

In order to examine our computations for the ordinary percolation with a continuous phase transition, we first consider the site percolation model on a square lattice of different sizes $L = 2^k, k = \{5, 6, \ldots, 12\}$. Each site can be either in an occupied or unoccupied state with probability p or $1-p$, respectively. All nearest-neighbor occupied sites will define a cluster assigned by a specified color. As a boundary condition, we fix all sites at the bottom boundary $(i, j = 1)$ in an occupied state which will be intact in time and constitutes the boundary domain (the cluster with the same color as the bottom-boundary occupied sites) whose statistical evolution is our main point of interest here. More precisely, to each boundary site $(i, j = 1)$ we attribute a height function h_i which is the maximum height of the occupied site in the column $i = 1, 2, \cdots, L$ belonging to the boundary domain (see Fig. 1). By running the occupancy p from 0 to 1, some occupied sites will randomly join to the boundary domain and thus the height profile $\{h_i\}$ will evolve as a function of p. The first quantity of interest is the height fluctuations measured by the root mean square (rms) w of the height profiles

$$\langle w\rangle_E = \left\langle \sqrt{\sum_i (h_i - \bar{h})^2/L} \right\rangle_E , \quad (1)$$

where $\bar{h}$ is the mean height and $\langle\cdots\rangle_E$ denotes for ensemble averaging. For a given occupancy p and system size L, the averages are taken over more than 5000 independent samples. We find that the width w exhibits a peak whose position converges to the site percolation threshold $p_c = 0.5927\ldots$ for large system sizes (see Fig. 2). At $p = p_c$, the boundary domain spans the lattice along the vertical direction. We also find that the value $\chi = w(p_c)$, which is called susceptibility, exhibits a scaling relation with the system size as $\chi \sim L^\gamma$. To estimate the exponent γ, the value of χ is averaged over 5×10^4 samples for each L, and we find that $\gamma = 0.997(3)$ (Fig. 3), very close to 1 in accord to the corresponding exponent for other percolation models even with discontinuous phase transition. We find the same exponent $\gamma \sim 1$ for the bond percolation model as well.

II. CLUSTER STATISTICS

In this section we present the results of our computations for the critical clusters of different rule models including the min-rule, max-rule, rnd-rule and the class of fractional percolation rules i.e., f-rule for $0 \le f \le 1$. The fractal dimensions of the critical clusters and their boundaries (or loops) are measured by examining the scaling relation between the average size s of the clusters, and the average length l of their boundaries with their average radius of gyration r_g, respectively (i.e., $s \sim r_g^{d_c}$ and $l \sim r_g^{d_l}$ where d_c and d_l denote for the fractal dimension of a critical cluster or its boundary, respectively–Figs. 4 and 5).

Figures 6 and 7 summarize the values of computed fractal dimensions for the critical clusters and their boundaries, respectively. Among them, the min-rule gives rise to compact clusters of dimension 2 with fractal boundaries while

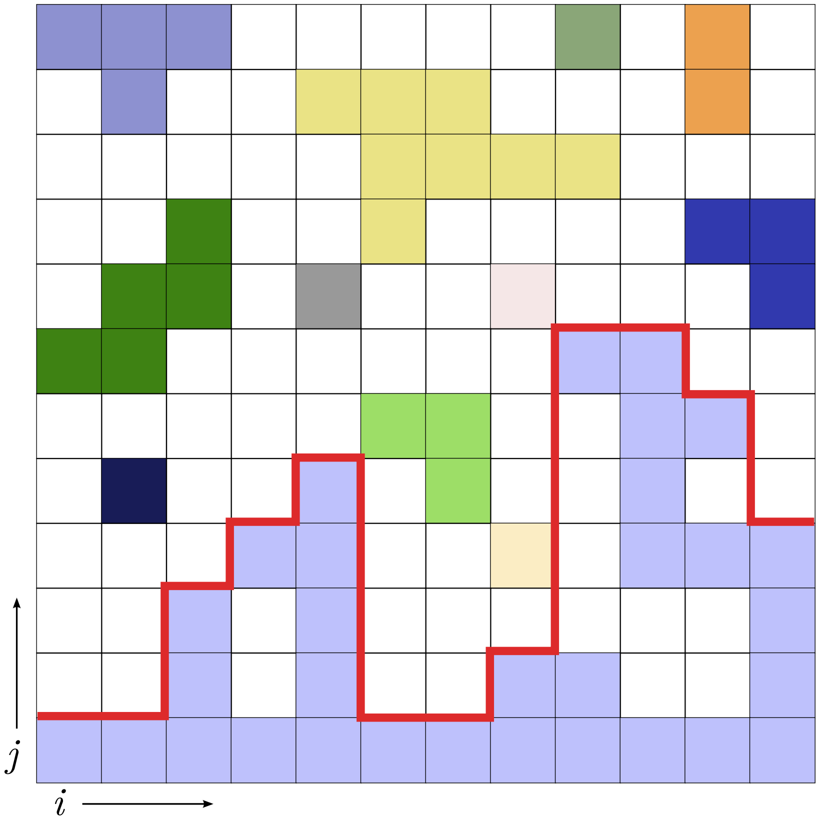

Figure 1: The boundary domain is the cluster of occupied sites attached to the bottom boundary sites which are fixed to be occupied as a boundary condition. The solid line shows the height profile attributed to each boundary site $(i, j = 1)$.

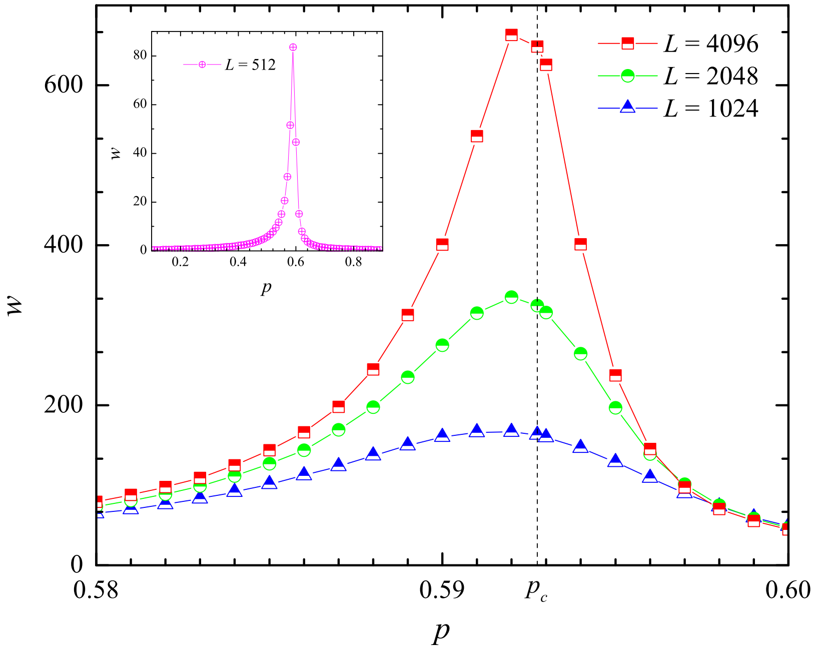

Figure 2: Main: The width w as a function of the occupancy p around p_c, for different system sizes L. Inset: w in the whole interval $p \in (0, 1)$.

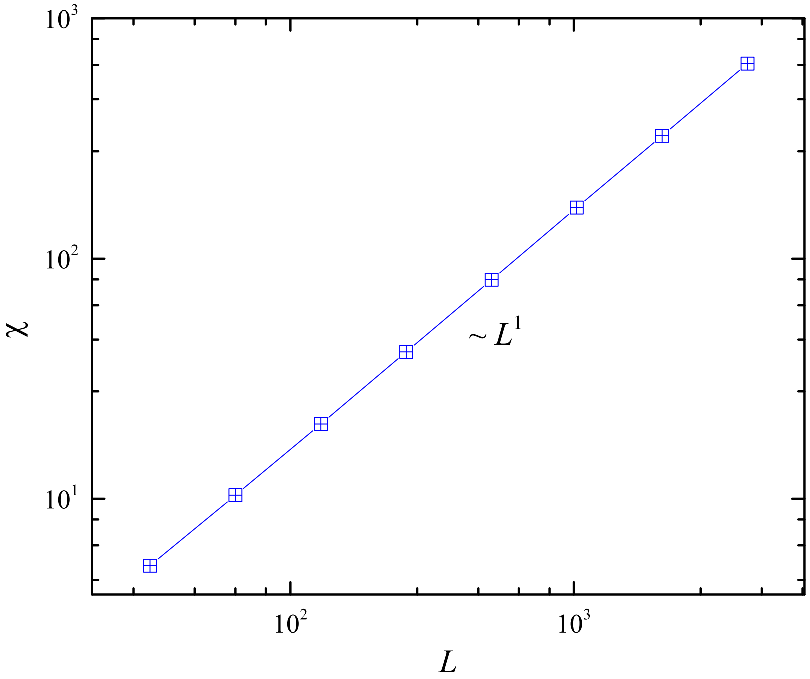

Figure 3: For the ordinary site percolation model, the susceptibility χ shows a scaling relation with the system size L, i.e., $\chi \sim L^{\gamma}$, with the exponent γ very close to 1.

Table I: The exponent τ for different rule models.

rule	max	$f = 0$
τ	1.79(2)	1.75(2)

for the other rules the critical clusters seem to have a porous structure. The other characteristic feature is that for different f-rules with $f > 0$, both fractal dimensions d_c and d_l seem to be f-independent within the error bars.

We have also examined the scaling relation $n_s \sim s^{-\tau}$, where n_s is the void size distribution in the spanning cluster at criticality for different rules. We find a conclusive scaling behavior only for the max-rule and f-rule with $f = 0$. For the other models, the spanning clusters are so compact either without or with a little average number of voids inside which elude a power-law behavior. The results are summarized in Table I.

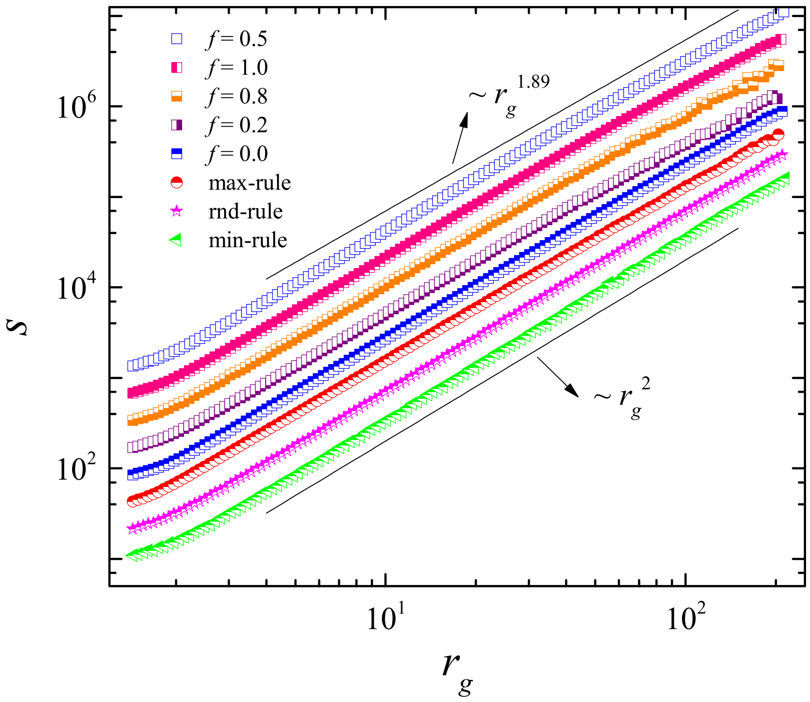

Figure 4: The average size s of critical clusters versus their average radius of gyration r_g for different rules obtained by averaging over 10^4 independent samples of size $L = 1024$.

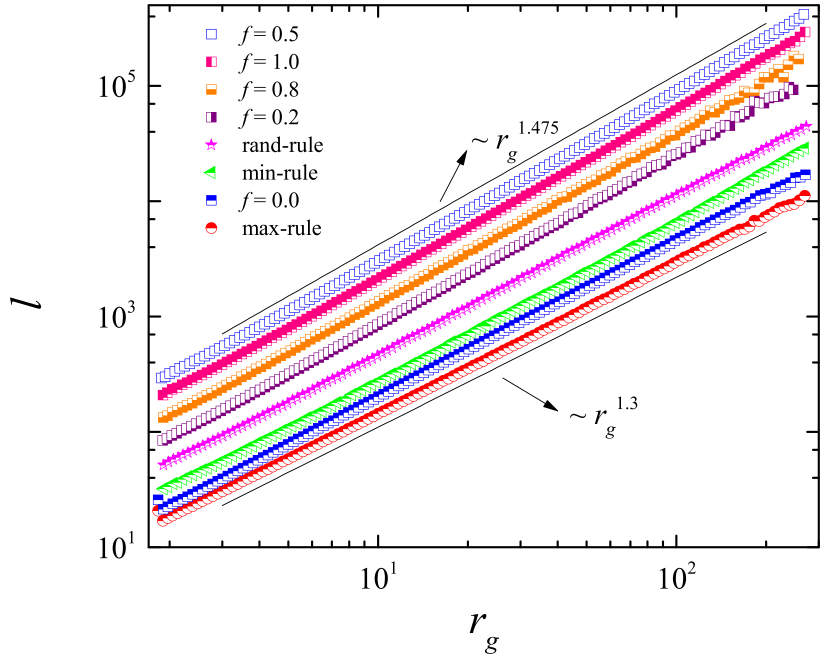

Figure 5: The average length l of the cluster boundaries versus their average radius of gyration r_g for different rules obtained by averaging over 10^4 independent samples of size $L = 1024$.

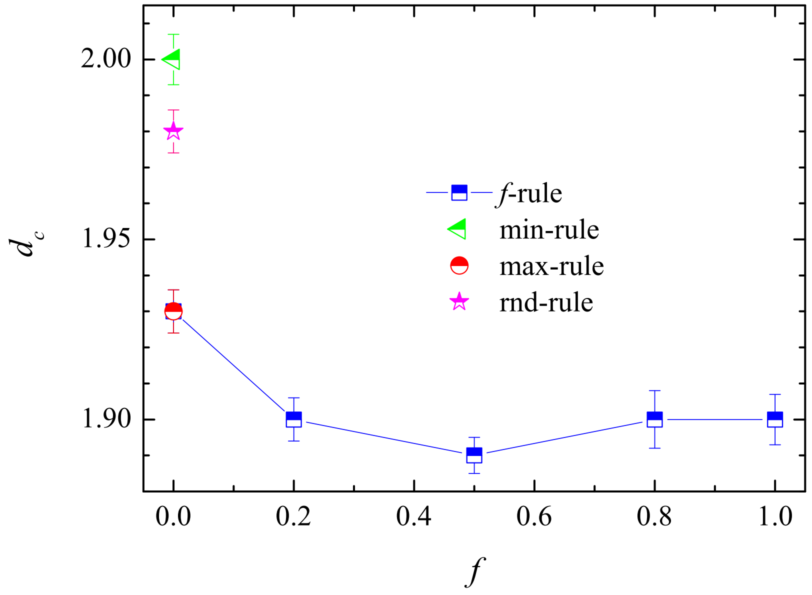

Figure 6: The fractal dimension d_c of a critical cluster for different rules.

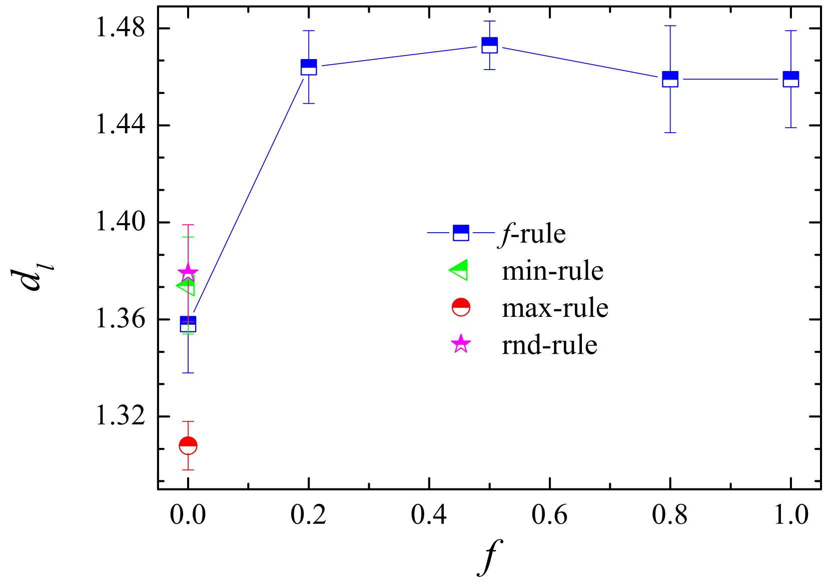

Figure 7: The fractal dimension d_l of a critical cluster boundary for different rules.
